# Prion-like protein gene (*PRND*) polymorphisms associated with scrapie susceptibility in Korean native black goats

**DOI:** 10.1371/journal.pone.0206209

**Published:** 2018-10-25

**Authors:** Min-Ju Jeong, Yong-Chan Kim, Byung-Hoon Jeong

**Affiliations:** 1 Korea Zoonosis Research Institute, Chonbuk National University, Iksan, Jeonbuk, Republic of Korea; 2 Department of Bioactive Material Sciences, Chonbuk National University, Jeonju, Jeonbuk, Republic of Korea; INRA Centre de Jouy-en-Josas, FRANCE

## Abstract

The polymorphisms of the prion protein (*PRNP*) gene, which encodes normal prion proteins (PrP), are known to be involved in the susceptibility of prion diseases. The prion-like protein (Doppel) gene (*PRND*) is the paralog of the *PRNP* gene and is closely located downstream of the *PRNP* gene. In addition, the polymorphisms of *PRND* correlate with disease susceptibility in several animals. We analyzed the genotype and allele frequencies of *PRND* polymorphisms in 246 Korean native black goats and found a total of six single nucleotide polymorphisms (SNPs) with one novel SNP, c.99C>T. We observed linkage disequilibrium (LD) within and between loci. *PRND* c.28T>C, c.151A>G, and c.385G>C and *PRND* c.65C>T and c.286G>A were in perfect LD and we have reported for the first time strong LD between *PRND* and *PRNP* or prion-related protein gene (*PRNT*) loci. Specifically, between the *PRND* c.28T>C, c.151A>G and c.385G>C and the *PRNP* codon 143, *PRND* c.99C>T and the *PRNP* codon 102 or *PRND* SNPs (c.28T>C, c.151A>G and c.385G>C) and *PRNT* SNP (c.321T>C). Furthermore, we confirmed that the genotype distribution of the *PRNP* p.His143Arg was significantly different according to that of the *PRND* c.28T>C (*P* < 0.0001). Finally, using PolyPhen-2 and PROVEAN, we predicted that two non-synonymous SNPs, c.65C>T and c.286G>A, in the *PRND* gene can have a detrimental effect on Doppel. To the best of our knowledge, this is the first report of genetic characteristics of the *PRND* gene in Korean native black goats.

## Introduction

Prion diseases, also called transmissible spongiform encephalopathies (TSEs), are notorious neurodegenerative diseases that include scrapie in sheep and goats, bovine spongiform encephalopathy (BSE) in cattle and Creutzfeldt–Jakob disease (CJD) in humans. The pathogenesis of prion diseases is associated with the aggregation of the deleterious prion protein (PrP^Sc^), which is converted from the benign prion protein (PrP^C^) [1,2].

Previous studies have reported that several polymorphisms of the prion protein gene (*PRNP*), which encodes PrP, can influence the susceptibility of prion diseases. Two polymorphisms of codons 129 and 219 in the human *PRNP* gene are considered crucial factors in determining susceptibility to human prion diseases [3–7]. Moreover, in small ruminants, such as sheep and goats, a number of polymorphisms associated with scrapie have been reported in the open reading frame (ORF) of the *PRNP* gene. Codons 136, 154 and 171 in the ovine *PRNP* gene are well known to be associated with the susceptibility to scrapie in sheep. In particular, by classifying the various haplotypes for three codons, including A_136_R_154_R_171_, V_136_R_154_Q_171_, and A_136_R_154_Q_171_, the disease-risk group of sheep could be estimated [1]. In goats, among 39 genetic variations, *PRNP* codons 127, 142, 143, 146, 154, 211, and 222 are known to contribute to the resistance to scrapie [8–21].

In recent studies, association studies of prion protein family genes have received attention as a novel view for prion diseases: prion-like protein gene (*PRND*), prion-related protein gene (*PRNT*), and shadow of prion protein gene (*SPRN*) which encode Doppel, Prt, and Shadoo, respectively [3,18,22,23]. Several polymorphisms in the paralogs of the *PRNP* gene have been shown to be associated with prion disease susceptibility [24–27]. The *PRND* gene is located downstream of the *PRNP* gene [28]. Previous studies have reported that two polymorphisms in the ORF and 3' untranslated region (UTR) +28 site of the *PRND* gene are associated with the progression of sporadic CJD in humans [26,29]. In sheep, the polymorphism at codon 26 of the *PRND* gene has been shown to correlate with disease susceptibility to scrapie and fertilization trait [27,30]. In goats, a study has been performed to identify prion disease-related SNPs of the *PRND* gene. However, since only 17 scrapie-affected animals were used in that study, the association between polymorphisms of the caprine *PRND* gene and scrapie was elusive [31].

Although the *PRND* gene has a significant relationship with prion disease susceptibility and reproductive ability, genetic studies of the caprine *PRND* gene have not been performed in Korean native black goats thus far. Here, we investigated the genotype, allele and haplotype frequencies of polymorphisms of the caprine *PRND* gene in 246 Korean native black goats. In addition, we performed an LD analysis among the single nucleotide polymorphisms (SNPs) of the *PRNP*, *PRND* and *PRNT* genes to find genetic linkage among the prion gene family. Furthermore, we predicted the possible impact of non-synonymous SNPs on the structure and function of the Doppel protein by using the algorithms PolyPhen-2 and PROVEAN.

## Materials and methods

### Ethical statement

All blood samples of the 246 Korean native black goats were purchased from a slaughterhouse in South Korea. All experimental processes were approved by the Chonbuk National University Institutional Animal Care and Use Committee (CBNU 2017–0076).

### Samples

Korean native black goats are the only Korean indigenous breed that has been farmed for over 2,000 years [32]. According to Statistics Korea (http://kostat.go.kr/portal/eng/index.action), the population of Korean native black goats is approximately 271,110 heads in 9,484 farms, and they are commonly used as meat and oriental medicine [32]. In addition, natural breeding without specialized artificial insemination has been practiced in Korean native black goats [32–34].

We obtained 246 blood samples of the Korean native black goats from a slaughter house, which provided goats from 8 farms in South Korea. The sample size used in this study may be enough to identify rare polymorphisms, including below 1% genotype frequency [35]. In addition, the sample size can also represent the total population of Korean native black goats with a 95% confidence level and a confidence interval of 7.

### Genetic analysis of the *PRND* gene

Genomic DNA was isolated from 200 μl of peripheral whole blood using the DNA Blood Mini Kit (Qiagen, Valencia, California, USA) following the manufacturer’s instructions. Polymerase chain reaction (PCR) was conducted using the following gene-specific sense and antisense primers: PRND-F (5’-TGCTCCAGCCTTTTCTGTTGCA-3’) and PRND-R (5’-CAGTGTGATTGATTCTTTAGCGC-3’). The PCR mixture was comprised of 2.5 μl of 10 × Taq DNA Polymerase buffer, 0.5 μl of 10 mM dNTP mixture, 1 μl each of sense and antisense primers, 2.5 μl of 5 × Band Helper, 0.2 μl of Taq DNA polymerase (Promega, Fitchburg, Wisconsin, USA), 1 μl of genomic DNA and sterile water to reach a total volume of 25 μl. The PCR cycling parameters were as follows: 95°C for 2 minutes, followed by 32 cycles of 95°C for 20 seconds, 59°C for 40 seconds, and 72°C for 1 minute, and then 1 cycle of 72°C for 5 minutes for final extension. PCR reaction was performed using a S-1000 Thermal Cycler (Bio-Rad, Hercules, California, USA). Amplified PCR products were purified using the Gel Extraction Kit (Qiagen, Valencia, California, USA) and sequenced with an ABI PRISM 3730XL Analyzer (ABI, Foster City, California, USA). Sequencing results were read using Finch TV software (Geospiza Inc, Seattle, USA), and genotyping was performed.

### Statistical analysis

The Hardy-Weinberg Equilibrium (HWE) test was applied to examine whether the random selection of the samples used in this study was well performed. The SNP Analyzer2.0 (http://snp.istech.info/istech/board/detail_snpa2.jsp) was used to conduct the HWE test and haplotype analysis. LD analysis was performed on all *PRND* SNPs by investigating Lewontin’s D’ (|D’|) and coefficient *r*^2^ using the program Haploview version 4.2 (Broad Institute, Cambridge, MA, USA).

### Analysis of the genetic linkage among SNPs of the *PRND*, *PRNP* and *PRNT* genes

LD analysis was performed among *PRNP*, *PRND* and *PRNT* SNPs. LD scores of the *PRNP* and *PRND* genes were calculated in 211 animals excepting 35 animals that did not have genotyping data for the *PRNP* gene. Next, the genotype distributions of *PRND* were compared with those of *PRNP*, and the distribution difference was calculated using the Chi-square test. All statistical analyses were calculated by Statistical Analysis Software (SAS), version 9.4 (SAS Institute Inc., Cary, NC, USA), and the statistically significant difference was determined by *P* value < 0.05.

### Prediction of the protein functional alteration by non-synonymous SNPs of the *PRND* gene

PolyPhen-2 and PROVEAN are *in silico* analysis tools that predict the impact of non-synonymous SNPs on the structure or function of a protein. PolyPhen-2 determines the impact of non-synonymous SNPs according to a position-specific, independent counts (PSIC) score difference. The results denote three types, “probably damaging”, “possibly damaging” and “benign”, depending on the degree of risk (http://genetics.bwh.harvard.edu/pph2/). PROVEAN evaluates the impact of non-synonymous SNPs by building up and comparing the clusters of related sequences and predicting the score. The results assign the term “deleterious” or “neutral” following a predefined threshold (e.g., -2.5) (http://provean.jcvi.org/seq_submit.php).

## Results

**Fig 1:**
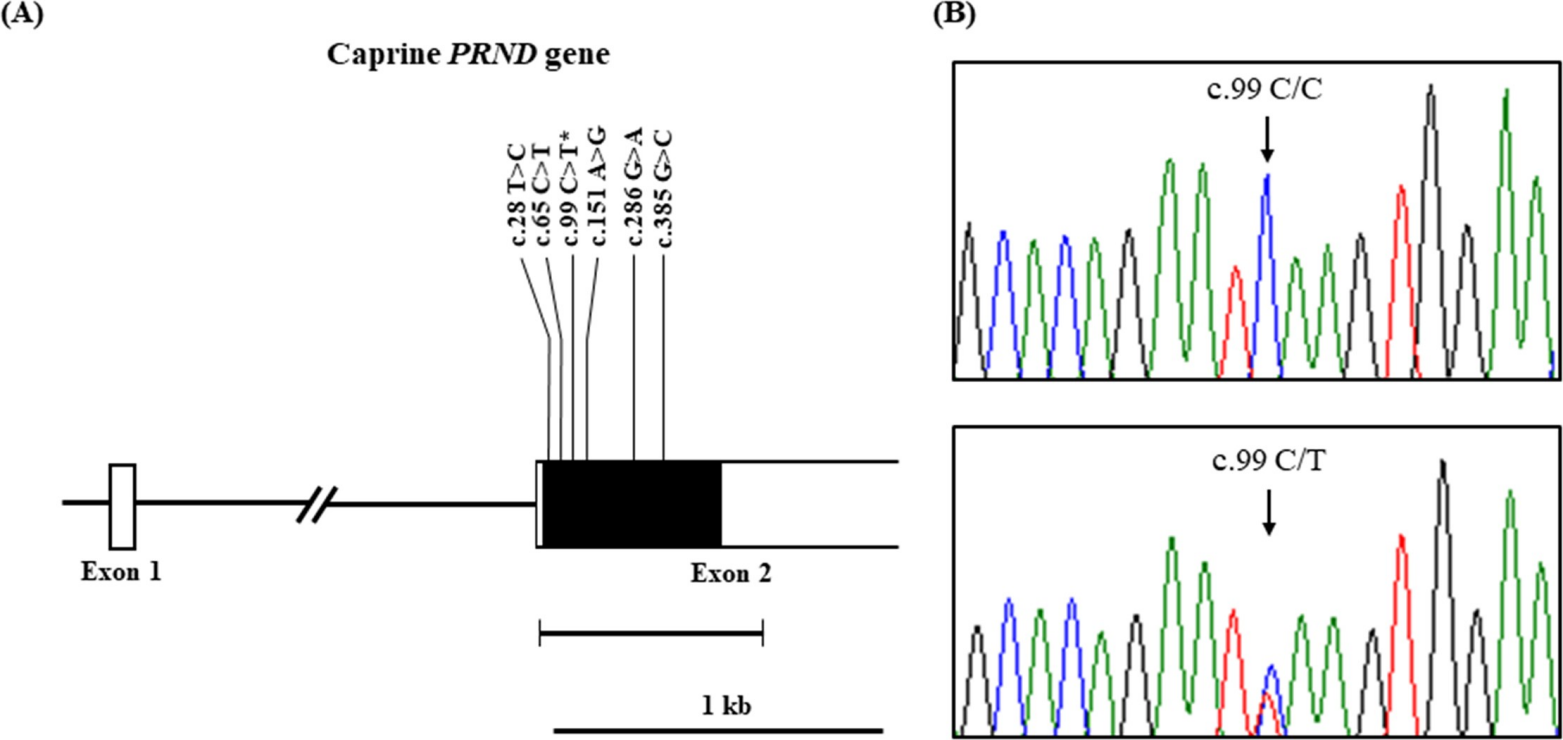
Genomic map and electropherograms of the single-nucleotide polymorphism (SNP) at c.99C>T of the caprine prion-like protein gene (*PRND*) in Korean native black goats. (A) Schematic diagram denotes the genomic structure of the caprine *PRND* gene, drawn to scale. The open reading frame (ORF) within exon 2 is indicated by the black box, and white boxes indicate the 5’ and 3’untranslated regions (UTRs). The edged horizontal bar indicates the regions sequenced. The bold text indicates the locations of the polymorphisms identified in this study. The asterisk indicates the novel SNP found in this study. (B) Electropherograms show two genotypes at c.99C>T of the caprine *PRND* gene in Korean native black goats. Upper panel, homozygote CC genotype; lower panel, heterozygote CT genotype. The homozygote TT genotype was not detected.

The caprine *PRND* gene is comprised of two exons and has a 537 bp ORF located in exon 2. We performed automatic direct sequencing on exon 2 of the caprine *PRND* gene and examined the genotype and allele frequencies of the *PRND* gene in 246 Korean native black goats. The DNA sequences in the current study are identical to that of the *PRND* gene of the *Capra hircus* registered in GenBank (Gene ID: 102170246). We found a total of six SNPs, including c.28T>C, c.65C>T, c.99C>T, c.151A>G, c.286G>A and c.385G>C, in the ORF of the caprine *PRND* gene (Fig 1A). Among them, four SNPs, c.65C>T (p.Ser22Phe), c.151A>G (p.Thr51Ala), c.286G>A (p.Glu96Lys) and c.385G>C (p.Val129Leu), are non-synonymous SNPs. The genotype and allele distribution of the caprine *PRND* gene is described in Table 1. The genotype frequencies of all SNPs were in accordance with HWE proportions.

**Table 1:** Genotype and allele frequencies of *PRND* polymorphisms in Korean native black goats.

	Genotype frequency, n (%)			Allele frequency, n (%)		HWE
c.28T>C	TT	TC	CC	T	C	
	91 (36.99)	121 (49.19)	34 (13.82)	303 (61.59)	189 (38.41)	0.535
c.65C>T	CC	CT	TT	C	T	
	230 (93.5)	16 (6.5)	0 (0)	476 (96.75)	16 (3.25)	0.598
c.99C>T	CC	CT	TT	C	T	
	233 (94.72)	13 (5.28)	0 (0)	479 (97.36)	13 (2.64)	0.670
c.151A>G	AA	AG	GG	A	G	
	91 (36.99)	121 (49.19)	34 (13.82)	303 (61.59)	189 (38.41)	0.535
c.286G>A	GG	GA	AA	G	A	
	230 (93.5)	16 (6.5)	0 (0)	476 (96.75)	16 (3.25)	0.598
c.385G>C	GG	GC	CC	G	C	
	91 (36.99)	121 (49.19)	34 (13.82)	303 (61.59)	189 (38.41)	0.535

Among the six SNPs, five were already registered on GenBank dbSNP (c.28T>C, rs668525432; c.65C>T, rs644252445; c.151A>G, rs657265876; c.286G>A, rs669682016; c.385G>C, rs645721044). However, we found one new SNP c.99C>T, and at this position, 94.72% were the homozygote CC genotype, and 5.28% were the heterozygote CT genotype (Fig 1B, Table 1).

We also investigated the extent of LD among the six SNPs of the caprine *PRND* gene by calculating the coefficient D’ and *r*^2^ values. All six SNPs were strongly linked together with a D’ value 1.0. However, the results using the *r*^2^ value indicated a weak LD for c.28T>C with c.65C>T, c.99C>T, and c.286G>A. The perfect LD (*r*^2^ score 1.0) is shown in c.28T>C, c.151A>G and c.385G>C as well as c.65C>T and c.286G>A (Table 2). In addition, we examined the haplotype frequency of these six *PRND* SNPs in Korean native black goats. As shown in Table 3, we detected the four haplotypes as follows: TCCAGG, CCCGGC, TTCAAG, and CCTGGC with frequencies of 58.3%, 35.8%, 3.3% and 2.6%, respectively.

**Table 2:** Linkage disequilibrium (LD) among six single nucleotide polymorphisms (SNPs) of the *PRND* gene in Korean native black goats.

	|D’|					
*r*^2^	c.28T>C	c.65C>T	c.99C>T	c.151A>G	c.286G>A	c.385G>C
c.28T>C	-	1.0	1.0	1.0	1.0	1.0
c.65C>T	0.017	-	1.0	1.0	1.0	1.0
c.99C>T	0.043	0.001	-	1.0	1.0	1.0
c.151A>G	1.0	0.017	0.043	-	1.0	1.0
c.286G>A	0.017	1.0	0.001	0.017	-	1.0
c.385G>C	1.0	0.017	0.043	1.0	0.017	-

**Table 3:** Haplotype frequencies of the six *PRND* polymorphisms in Korean native black goats.

Haplotype	Korean native black goats (n = 246)
TCCAGG	287 (0.583)
CCCGGC	176 (0.358)
TTCAAG	16 (0.033)
CCTGGC	13 (0.026)

**Fig 2:**
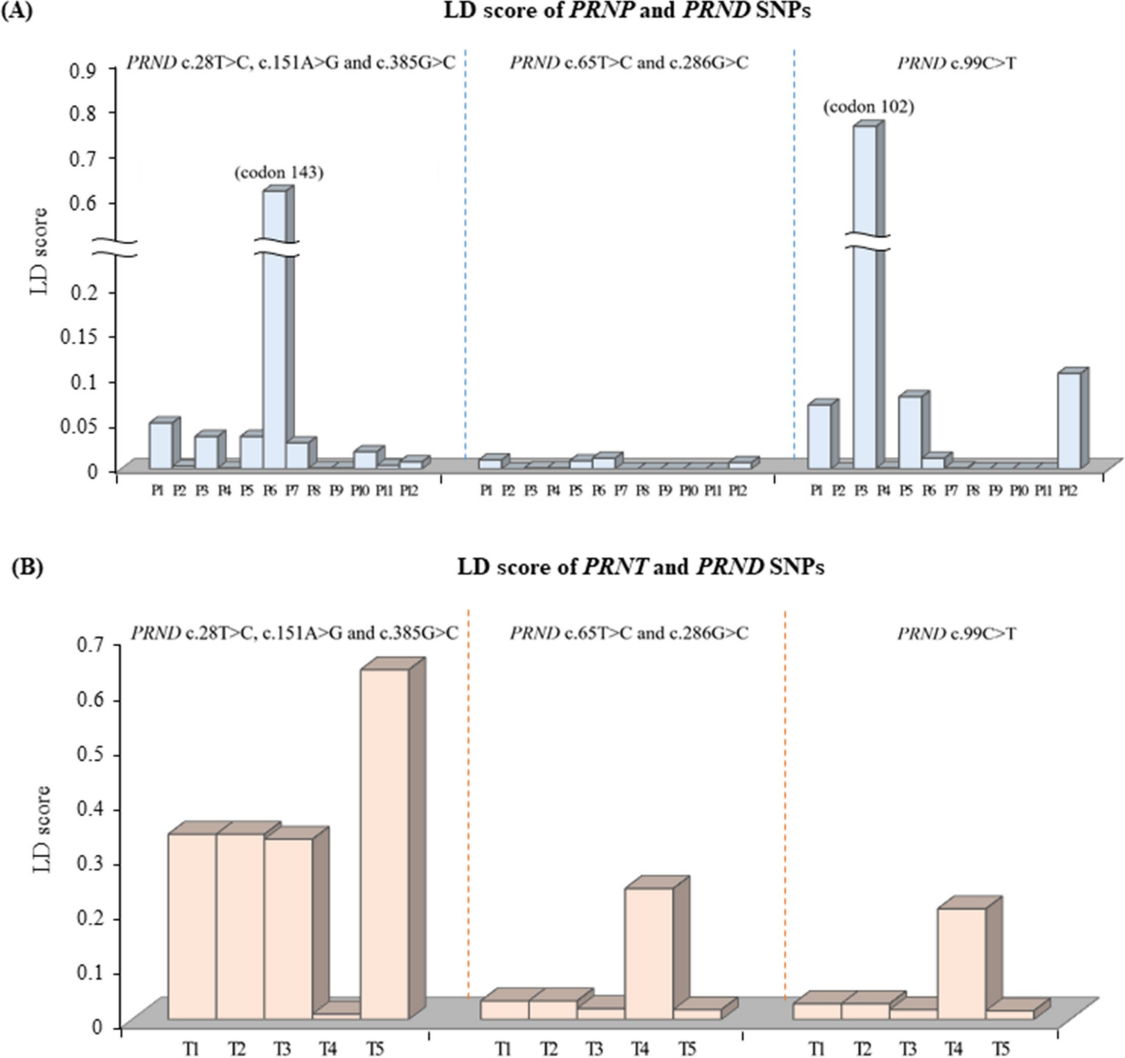
The linkage disequilibrium (LD) scores between single nucleotide polymorphisms (SNPs) of the *PRND* gene and those of the *PRNP* and *PRND* genes. (A) The LD scores between *PRND* and *PRNP* SNPs. P1 ~ P12 indicate *PRNP* SNPs as follows: P1, c.126G>A (codon 42); P2, c.302A>G (codon 101); P3, c.304T>G (codon 102); P4, c.379G>A (codon 127); P5, c.414T>C (codon 138); P6, c.428A>G (codon 143); P7, c.437A>G (codon 146); P8, c.461G>A (codon 154); P9, c.512A>G (codon 171); P10, c.632G>A (codon 211); P11, c.652A>C (codon 218); P12, c.718C>T (codon 240). (B) The LD scores between *PRND* and *PRNT* SNPs. T1 ~ T5 indicate *PRNT* SNPs as follows: T1, c.-114G>T; T2, c.-58A>G; T3, c.71C>T (codon 24); T4, c.102G>A; T5, c.321T>C.

To investigate whether caprine *PRND* gene polymorphisms have genetic linkage to polymorphisms of the *PRNP* gene, we carried out LD analysis between polymorphisms of the *PRNP* and *PRND* genes with *r*^2^ values (Fig 2A). Detailed values of LD analysis are described in S1 Table. A group including *PRND* c.28T>C, c.151A>G and c.385G>C has a strong LD with only *PRNP* codon 143 SNP (*r*^2^ value: 0.612). Another group including *PRND* c.65C>T and c.286G>A has a weak LD value below 0.05 scores in all 12 *PRNP* SNPs. *PRND* c.99C>T has a strong LD with only *PRNP* codon 102 SNP (*r*^2^ value: 0.78).

We also investigated the genetic linkage between polymorphisms of the *PRND* and *PRNT* genes (Fig 2B and S2 Table). A group including *PRND* c.28T>C, c.151A>G and c.385G>C has a strong LD with *PRNT* c.321T>C (*r*^2^ value: 0.638). Another group including *PRND* c.65C>T, c.286G>A and c.99C>T has a weak LD value below 0.25 scores in all 5 *PRNT* SNPs.

**Fig 3:**
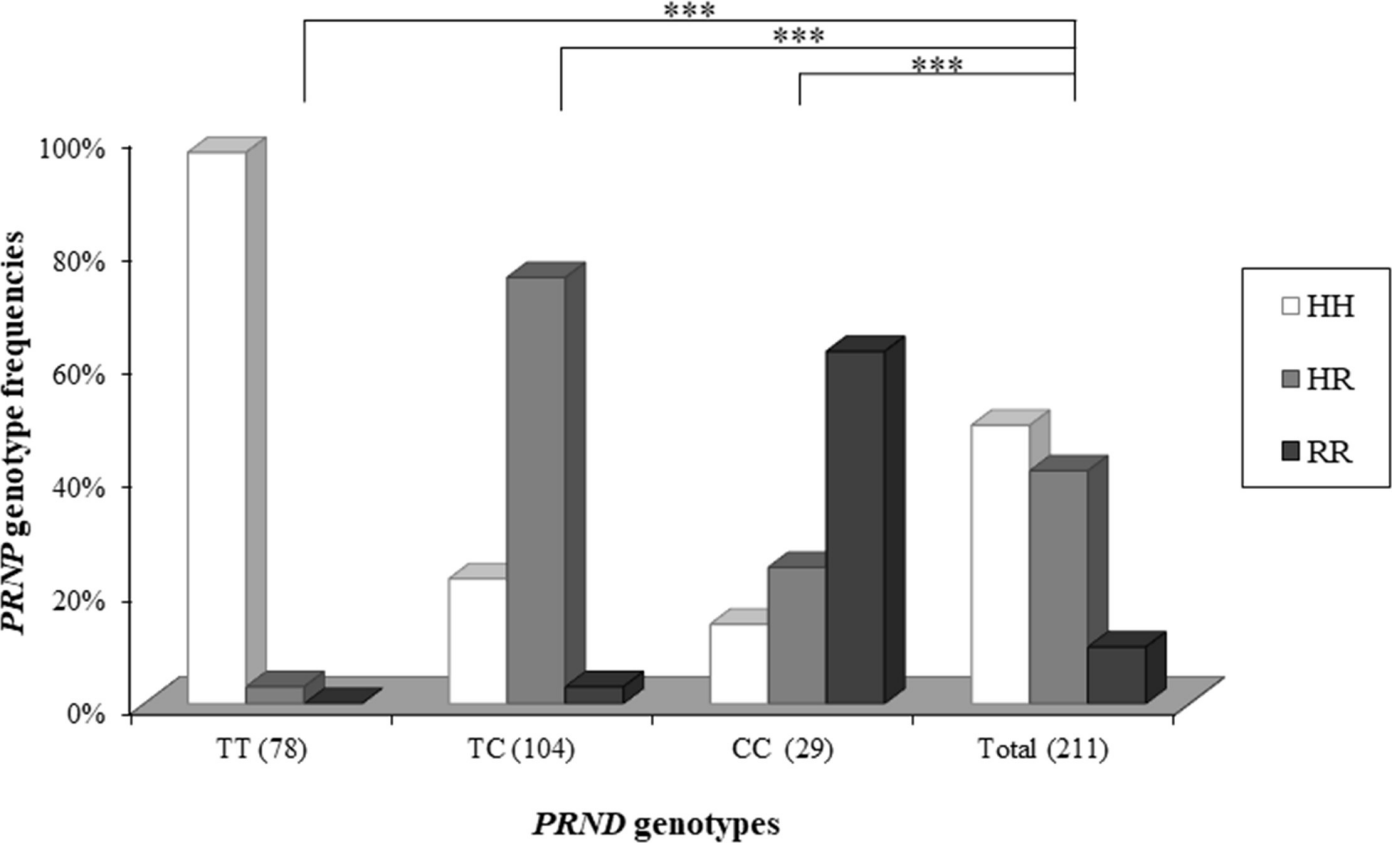
Genotype distribution of *PRNP* p.His143Arg (*PRNP* c.428A>G) according to genotypes in *PRND* c.28T>C. The *P* value indicates a significant difference in the *PRNP* genotype distribution compared to that of the total population. *** *P* < 0.0001.

In addition, to confirm the combined effects of the *PRND* and *PRNP* genes, we investigated the genotype distribution of *PRNP* p.His143Arg according to the genotype distributions of *PRND* c.28T>C (Fig 3). Compared to the general distribution of the *PRNP* p.His143Arg, the genotype distribution of *PRNP* p.His143Arg according to that of *PRND* c.28T>C is significantly different in all *PRND* genotypes (*P* < 0.0001) (Fig 3). Notably, the *PRNP* HH genotype accounts for the highest distribution in the *PRND* TT genotype (97.4%). In addition, the *PRNP* HR genotype makes up the highest distribution in the *PRND* TC genotype (75%), and the *PRNP* RR genotype is the highest in the *PRND* CC genotype (62.1%).

To assess the potential damaging impact of non-synonymous SNPs in the caprine *PRND* gene, we utilized PolyPhen-2 and PROVEAN. PolyPhen-2 predicted c.65C>T (p.Ser22Phe) as ‘Possibly damaging’ with a score of 0.951. PROVEAN predicted c.286G>A (p.Glu96Lys) as ‘Deleterious’ with a score of 2.705 (Table 4).

**Table 4:** Functional prediction of non-synonymous single nucleotide polymorphisms (SNPs) in Korean native black goats by PolyPhen-2 and PROVEAN.

Variations	PolyPhen-2		PROVEAN	
	Score	Prediction	Score	Prediction^a^
c.65C>T (p.Ser22Phe)	0.951	Possibly damaging	-2.494	Neutral
c.151A>G (p.Thr51Ala)	0.049	Benign	-0.487	Neutral
c.286G>A (p.Glu96Lys)	0.114	Benign	-2.705	Deleterious
c.385G>C (p.Val129Leu)	0.004	Benign	-1.465	Neutral

^a^ PROVEAN prediction cutoff = -2.5

## Discussion

Because of the close genomic location and similar structure to *PRNP*, *PRND* has been noted as another major candidate gene in prion diseases [3,18,26]. Previous studies have reported the relationship of prion diseases with the *PRND* gene in a broad spectrum of hosts, including humans, cattle, sheep and goats [26,31,36–41]. Therefore, it is important to verify the genetic characteristics of the *PRND* gene in Korean native black goats. Here, we performed direct sequencing in 246 Korean native black goats and carried out genotyping. We found six polymorphisms, including one novel SNP, *PRND* c.99C>T. Moreover, perfect LD scores were observed in *PRND* c.28T>C, c.151A>G, and c.385G>C; *PRND* c.65C>T and c.286G>A with an *r*^2^ value of 1.0.

In a previous study, it was demonstrated that a strong genetic linkage existed in scrapie-associated SNPs between the *PRNP* and *PRND* genes in sheep [27]. Because goats are another major host of scrapie, we searched for such genetic linkage in the scrapie-associated SNPs among the *PRNP*, *PRND* and *PRNT* genes in Korean native black goats. For this, we investigated the genetic linkage between the SNPs of two genes by calculating the *r*^2^ value. A group including *PRND* c.28T>C, c.151A>G, and c.385G>C is genetically linked to *PRNP* codon 143 (Figs 2 and 3). These data reveal that the *PRND* TT genotype showed genetically involved distribution with the *PRNP* HH genotype. However, the *PRNP* codon 143 SNP (with Arg instead of His) has been revealed to have a relatively weak influence on scrapie progression compared to two other SNPs at codon 146 (Asp or Ser, instead of Asn) and codon 222 (Lys instead of Gln) [9–11,18,42–45]. Therefore, the genetic linkage should be further investigated to determine how it can affect the progression of prion disease. In addition, the strong genetic linkage between *PRND* and *PRNT* genes identified in the present study may be highly helpful in later reproductive studies since the genes have been shown to be testis-specific and related to spermatogenesis [46,47].

Finally, we evaluated the possible effect of four non-synonymous SNPs on Doppel using PolyPhen-2 and PROVEAN. Notably, c.65C>T (p.Ser22Phe) and c.286G>A (p.Glu96Lys) are damaging to Doppel (Table 4). Previous studies were mainly performed to examine the association between polymorphisms of the *PRND* gene and prion disease susceptibility. However, because Doppel protein was mainly expressed in a testis-specific manner, *PRND*-knockout mice resulted in infertility due to interference of sperm-egg interaction [48]. Furthermore, a recent study has reported that the genotype of the *PRND* gene affects the capacitation process and reproductive power of the spermatozoa in ram [46]. Therefore, additional functional studies are needed on the fertility and disease susceptibility of these non-synonymous SNPs in the caprine *PRND* gene.

In conclusion, we found a total of six SNPs, including a novel SNP, *PRND* c.99C>T, through direct sequencing of the *PRND* gene in 246 Korean native black goats. In addition, we reported strong genetic linkage among *PRNP*, *PRND* and *PRNT* SNPs in goats. Finally, we annotated four non-synonymous SNPs of the caprine *PRND* gene using PolyPhen-2 and PROVEAN and predicted that two non-synonymous SNPs (c.65C>T and c.286G>A) are deleterious to the Doppel protein. To our knowledge, this is the first genetic study of the *PRND* gene in Korean native black goats.

## Supporting information

S1 Table Linkage disequilibrium (LD) between *PRNP* and *PRND* SNPs with *r*^2^ values in Korean native black goats. (PDF)

S2 Table Linkage disequilibrium (LD) between *PRND* and *PRNT* SNPs with *r*^2^ values in Korean native black goats. (PDF)
