# Supplementary material for: Prion-like protein gene (PRND) polymorphisms associated with scrapie susceptibility in Korean native black goats
Source: PLoS One. 2018 Oct 25;13(10):e0206209. doi: 10.1371/journal.pone.0206209 (PMC6201918; doi:10.1371/journal.pone.0206209)
Supplement: S1 Table — (PDF) [file pone.0206209.s001.pdf]

S1 Table. Linkage disequilibrium (LD) between *PRNP* and *PRND* SNPs with r^2 value in Korean native black goats.

	PRND					
PRNP	c.28T>C	c.65C>T	c.99C>T	c.151A>G	c.286G>A	c.385G>C
c.126G>A (codon 42)	0.051	0.01	0.071	0.051	0.01	0.051
c.302A>G (codon 101)	0.003	0	0	0.003	0	0.003
c.304T>G (codon 102)	0.036	0.001	0.78	0.036	0.001	0.036
c.379G>A (codon 127)	0.001	0.001	0.001	0.012	0.001	0.012
c.414T>C (codon 138)	0.036	0.009	0.08	0.036	0.009	0.036
c.428A>G (codon 143)	0.612	0.012	0.012	0.612	0.012	0.612
c.437A>G (codon 146)	0.029	0	0.001	0.029	0	0.029
c.461G>A (codon 154)	0.001	0	0	0.001	0	0.001
c.512A>G (codon 171)	0.001	0	0	0.001	0	0.001
c.632G>A (codon 211)	0.019	0	0	0.019	0	0.019
c.652A>C (codon 218)	0.004	0	0	0.004	0	0.004
c.718C>T (codon 240)	0.008	0.007	0.106	0.008	0.007	0.008
