# Supplementary material for: Prion-like protein gene (PRND) polymorphisms associated with scrapie susceptibility in Korean native black goats
Source: PLoS One. 2018 Oct 25;13(10):e0206209. doi: 10.1371/journal.pone.0206209 (PMC6201918; doi:10.1371/journal.pone.0206209)
Supplement: S2 Table — (PDF) [file pone.0206209.s002.pdf]

S2 Table. Linkage disequilibrium (LD) between *PRND* and *PRNT* SNPs with r^2 value in Korean native black goats.

	PRND					
PRNT	c.28T>C	c.65C>T	c.99C>T	c.151A>G	c.286G>A	c.385G>C
c.-114G>T	0.338	0.034	0.029	0.338	0.034	0.338
c.-58A>G	0.338	0.034	0.029	0.338	0.034	0.338
c.71C>T (codon 24)	0.329	0.002	0.018	0.329	0.002	0.329
c.102G>A	0.01	0.239	0.202	0.01	0.239	0.01
c.321T>C	0.638	0.018	0.016	0.638	0.018	0.638
